# 586. Immunogenicity of COVID-19 mRNA Vaccines in Patients with Lymphoid Malignancies

**DOI:** 10.1093/ofid/ofab466.784

**Published:** 2021-12-04

**Authors:** Natalie E Izaguirre, Amy C Sherman, Jennifer Crombie, Michaël Desjardins, Chi-An Cheng, Tal Gilboa, Megan Powell, Bruce P Bausk, Noah Abasciano, Peter Baker, Mikaela McDonough, Philippe Armand, David Walt, Nicolas C Issa, Lindsey R Baden

**Affiliations:** 1 Brigham and Women's Hospital, Boston, Massachusetts; 2 Harvard Medical School/Brigham and Women's Hospital, Boston, Massachusetts; 3 Dana Farber Cancer Institute, Boston, Massachusetts; 4 Brigham and Women’s Hospital, Boston, Massachusetts; 5 Brigham and Womens' Hospital, Brookline, Massachusetts; 6 BWH Division of Infectious Diseases, Boston, Massachusetts; 7 Brigham And Women's Hospital, Hampton, New Hampshire; 8 Dana-Farber Cancer Institute, Boston, Massachusetts; 9 Harvard Medical School/Brigham and Women's Hospital/Wyss Institute, Boston, Massachusetts

## Abstract

**Background:**

Patients with lymphoid malignancies are at high risk of severe COVID-19 disease and were not included in the phase 3 mRNA vaccine trials. Many patients with lymphoid malignancies receive immunosuppressive therapies, including B-cell depleting agents, that may negatively impact humoral response to vaccination.

**Methods:**

We recruited patients with lymphoid malignancies and healthy participants who planned to receive two doses of SARS-CoV-2 mRNA vaccine (BNT162b2 or mRNA-1273). Blood was drawn at baseline, prior to second dose of vaccine, and 28 days after last vaccination. Disease characteristics and therapies were extracted from patients’ electronic medical record. An ultrasensitive, single molecule array (Simoa) assay detected anti-Spike (S), anti-S1, anti-receptor binding domain (RBD), and anti-Nucleocapsid (N) IgG from plasma at each timepoint.

**Results:**

23 healthy participants and 37 patients with lymphoid malignancies were enrolled (Table 1). Low titers of anti-N (Fig 1A) demonstrate no prior exposure or acquisition of COVID-19 before vaccination or during the study. 37.8% of the lymphoid malignancy cohort responded to the vaccine, using an internally validated AEB cutoff of 1.07. A significantly higher magnitude of anti-S (p< 0.0001), anti-S1 (p< 0.0001) and anti-RBD (p< 0.0001) are present in the healthy as compared to lymphoid malignancy cohort at the second dose and day 28 post-series (Fig 1B, Fig 1C and Fig 1D). Anti-S IgG titers were compared between the healthy cohort, treatment naïve, and treatment experienced groups (Fig 2). The treatment naïve cohort had high titers by series completion which were not significantly different from the healthy cohort (p=0.2259), although the treatment experienced group had significantly decreased titers (p< 0.0001). Of the 20 patients who had received CD20 therapy, there was no clear correlation of anti-S IgG response with time from CD20 therapy, although most patients who received CD20 therapies within 12 months from the vaccine had no response (Figure 3).

Table 1. Demographics

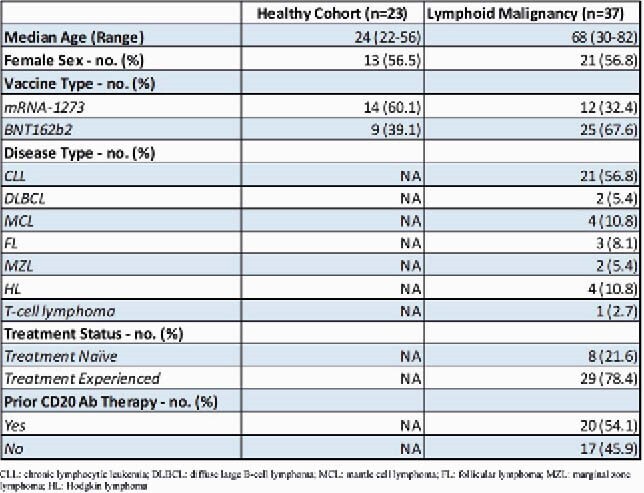

Figure 1. Anti-N, Anti-S, Anti-S1, Anti-RBD and Anti-N Ig G for healthy v. lymphoid malignancy cohort

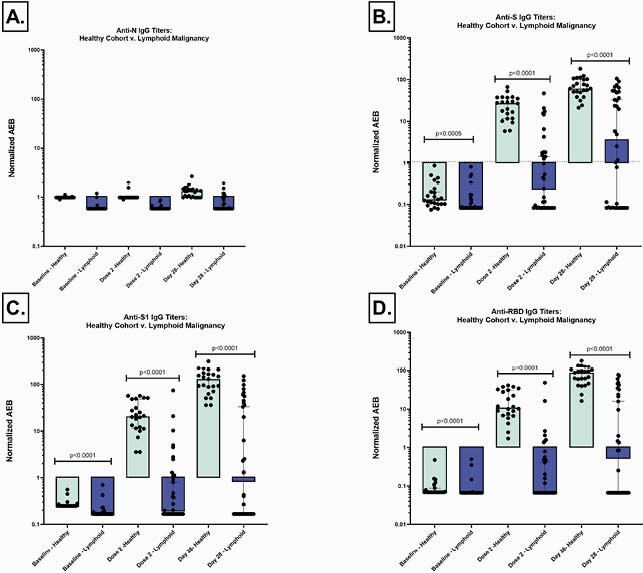

The dotted line at 1.07 marks in an internally validated threshold to mark anti-S IgG response. The black bars denote median with 95% CI.

Figure 2: Anti-S IgG for healthy v. treatment naïve v. treatment experienced

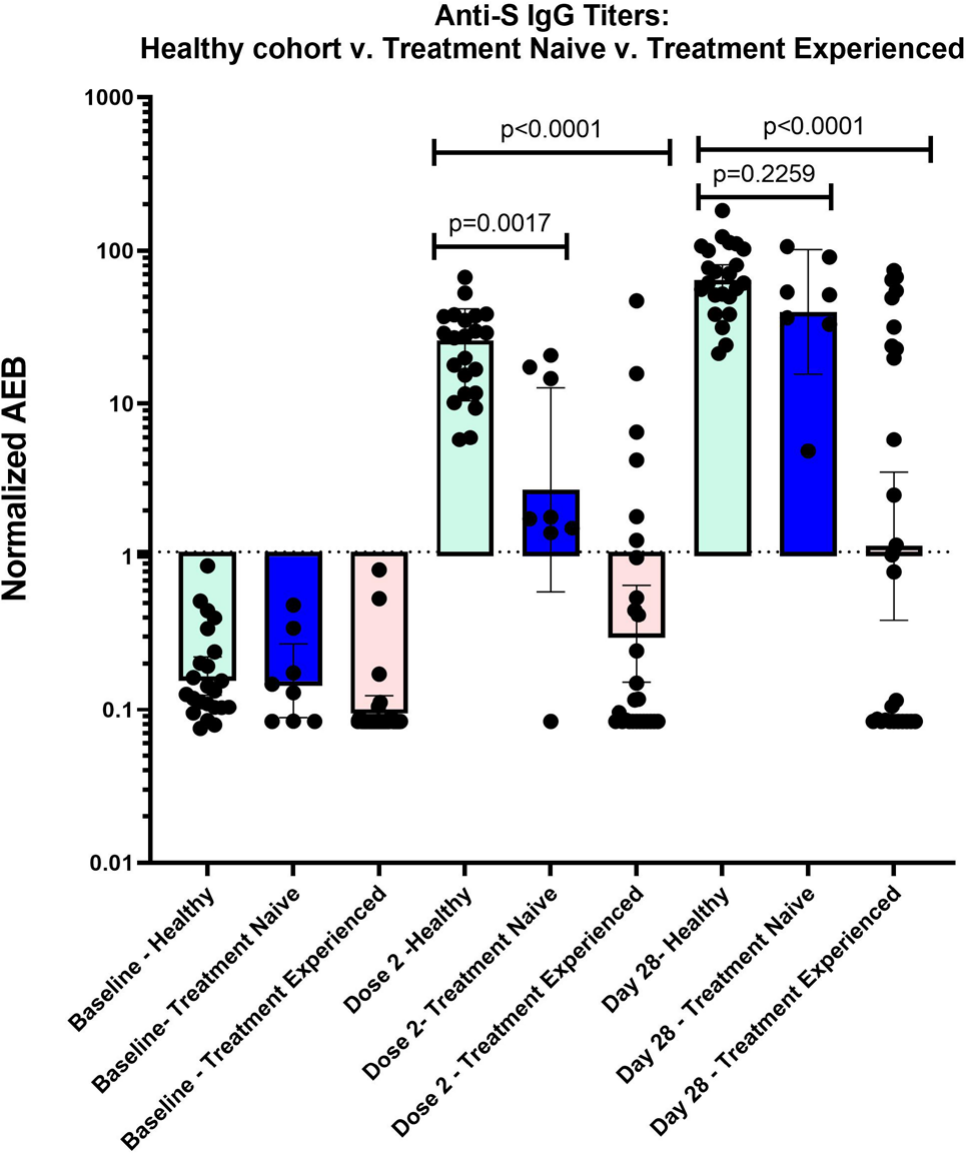

The dotted line at 1.07 marks in an internally validated threshold to mark antibody response. The black bars denote median with 95% CI.

**Conclusion:**

The vaccine-induced immune response was poor among treatment-experienced patients with lymphoid malignancies, especially among those who received CD20 therapies within 12 months.

Figure 3. Months from CD20 therapy v. anti-S IgG titers

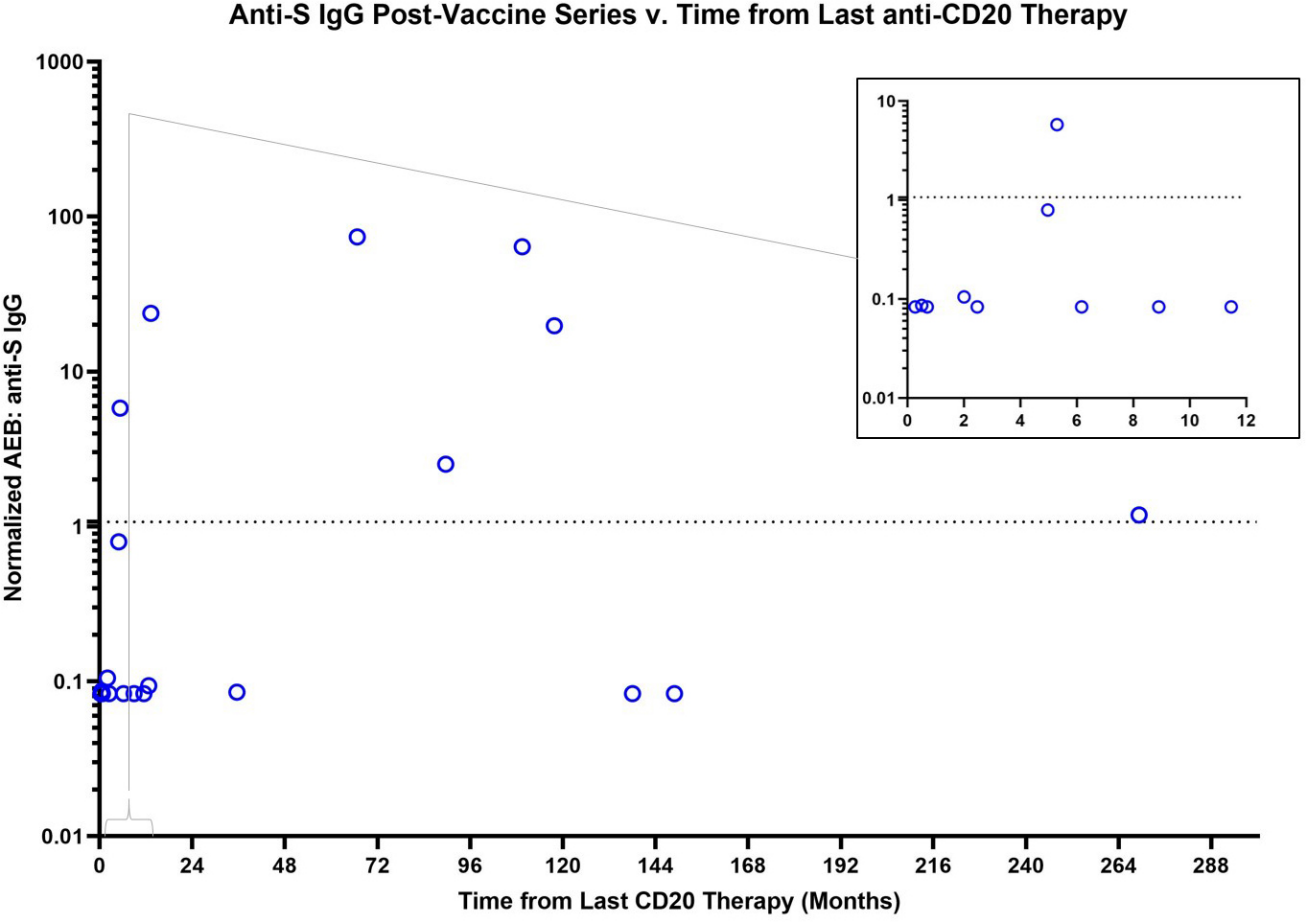

The dotted line at 1.07 marks in an internally validated threshold to mark antibody response.

**Disclosures:**

**Jennifer Crombie, MD**, **AbbVie** (Grant/Research Support)**Bauer** (Grant/Research Support)**Karyopharm** (Consultant)**MorphoSys** (Consultant) **Philippe Armand, MD PhD**, **ADCT, Celgene, Morphosys, Daiichi, Miltenyi, Tessa, C4, Genmab, Enterome, Regeneron, Genentech, Epizyme, Astra Zeneca** (Consultant, Sorry to put them all in, hope you can deconvolute for me)**Affimed, Adaptive, BMS, Merck, Kite, IGM, Genentech** (Research Grant or Support, Institutional research funding) **David Walt, PhD**, **Quanterix Corporation** (Board Member, Shareholder) **Nicolas C. Issa, MD**, **AiCuris** (Scientific Research Study Investigator)**Astellas** (Scientific Research Study Investigator)**GSK** (Scientific Research Study Investigator)**Merck** (Scientific Research Study Investigator)

